# The Medical Society of London

**Published:** 1889-06

**Authors:** 


### THE MEDICAL SOCIETY OF LONDON.

The annual *conversazione* of the Medical Society of London was held in the Society's rooms on May 6th; the Fellows and guests were received by the President, Dr. C. Theodore Williams, immediately after the annual Oration had been delivered by Mr. Jonathan Hutchinson. Some of the instantaneous photographs, illustrating consecutive phases of locomotion, made by Mr. Eadweard Muybridge, of the University of Pennsylvania, were exhibited. Dr. C. Theodore Williams showed Ketchum's pneumatic cabinet, and Mr. Edmunds the graphophone.

Mr. Jonathan Hutchinson chose for the topic of his lecture "The Clinical Uses of Rare Diseases." The diseases which presented the most apparent peculiarity were often, if correctly understood, the most definite examples of evolution under ordinary laws. Of a very large number of diseases which ranked as rare it must be asserted that they were simply exaggerated or over-grown examples of those which were common. They presented nothing really novel, but showed everything strongly marked. Not the most superficial observer could fail to have his attention arrested by a well characterized example of Graves' disease; but for one case which rises to such degree of severity as to justify the diagnosis, there were probably twenty in which the condition was threatened, but never became complete. Further, there was no law under which Graves' malady necessarily acquired its full characteristics; so far from its being a malady *sui generis*, it was only a name given to extreme examples of a certain disturbance of vasomotor function of which minor instances were of everyday occurrence. Here the common might help the rare, as offering illustrations of treatment; for after much indefatigable search for drugs which might control the locally defective vasomotor function, the conclusion seemed to be that the real remedy in all cases, the severe as well as the slight, was a prolonged and well-marked change of climate; again, it would be admitted even more readily than in the case of Graves' disease that the domain of myxœdema was not well defined. For one case that was well marked there were twenty that were not so. So common, indeed, were some of its features—the flabby, thickened skin, the featureless face, the general obtunding of intellect—that we might be tempted to suspect that it was only one of the ordinary forms of premature senility to which a large proportion of women were liable.

If it were true that many of the diseases accounted rare were simply exaggerated specimens of what was common, it was also true of others that they revealed individual idiosyncrasies of exaggerated intensity. The malady known as xeroderma pigmentosum (or perhaps preferably after the name of the first observer, "Kaposi's disease") was a noteworthy instance of this; all examples of unusual freckling, indeed, perhaps all freckling whatever, denoted a minor form of Kaposi's disease. In rare cases, and chiefly in connection with marked family tendency (as made evident by several brothers and sisters being successively attacked), the proclivity might go much further. The freckles might coalesce, might inflame, ulcerate, destroy the features, produce neoplasms, and even pass on into epithelial cancer, yet, in scleroderma pigmentosum there was no pathological novelty whatever. It was very far from being a disease *sui generis.* It was simply an exaggeration of what was common, receiving its final intensification under those laws of heredity which evoked "family" diseases. Raynaud's disease afforded a good illustration of the doctrine of exaggeration as explanatory of rare diseases. Every one who got a dead finger from putting his hands too long in cold water, or a blue nose from exposure to a wintry wind, illustrated in those occurrences all that was essential to the malady in question. An exaltation of reflexes which were normal, and intensification of vasomotor susceptibility which in low degree were a part of the organization of the most healthy, such was all that was witnessed in the most marked forms of Raynaud. Finally, as had been pointed out by Dr. Barlow, the conditions which were at first functional only and transitory, might gradually produce structural changes, and the case which was simply asphyxia of the extremities at first, might slide on into one of diffuse scleriasis of the skin.

As illustrations of what might be learnt in reference to anatomical and physiological laws from the careful observation of rare cases, he would mention three examples of a demonstration as to the position of the spinal center for the act of copulation; physiologists, indeed, were agreed that it was higher than, and distinct from, that which supplied muscular force to the sphincters and regulated micturition and defæcation. A gentleman who had recovered after a severe fracture of the lower dorsal vertebræ, with considerable displacement, had permanent paralysis of the sphincters of the anus and urethra, and some weakness of both the lower extremities. He retained the power of copulation undiminished. In two very parallel cases, young gentlemen who had experienced spontaneous recovery from spina bifida applied to him for leave to marry. Both were quite confident as to their sexual ability, and both were now happily married. Yet in both of them the two sphincters were paralyzed. It was of great importance to make good use of all opportunities which daily practice afforded for observation as to physiological laws of this kind.

In the remarkable affection recently named "dry mouth," or xerostomia, the whole mouth became quite dry, and, since the condition was unattended by any other symptoms, it might be taken as proved that there was a center which controlled the function at once of the salivary glands and of all the mucous crypts and smaller gland organs which were found in the mucous membrane of the cheeks, palate, gums, and tongue.

It was not unimportant to assert that there were absolutely no cases which were unique. However strange and bizarre might be the phenomena in any individual case, we had but to wait long enough to find their exact parallel. This fact proved that ordinary laws underlay all that we recognize by the name of disease, however exceptional. There was a form of epithelial cancer of the face—crateriform ulcer—which was like nothing else, and all the examples of it were exactly alike. There must be some very special influence which stamped the exceptional disease (the crateriform) in all its stages with such peculiar features of individuality. He had noticed the conjunction of what might be called the black freckles of the aged with cancer. A gentleman near 60 years of age came with an ugly fungating ulcer in his lower eyelid. It was clearly malignant, below it was a coal-black patch, which had been there for six years or more, and was gradually increasing. The malignant ulcer close to it had been present only about six months. Within a few months another case, in all respects exactly like, came under his care. In this instance the patient was an elderly lady. It seemed difficult to withstand the suggestion that there must be some connection between the black patches and the cancer. The close juxtaposition of the two conditions could scarcely be a mere coincidence. He believed that they illustrated, in the first place, the general law that, in aged persons, all local disturbances of nutrition had a tendency to slide on into cancerous action; and next another law, as regards malignancy, that its precise form might change with the precise tissue attacked.

In connection with these extremely rare examples of malignant development in senile structures, and of the gradual transformation of innocent hypertrophies into malignant ones, he instanced granuloma fungoides: the essential feature of the disease was that the skin was attacked in the first instance by areas of inflammatory thickening, having no similarity to cancer in any way. Gradually the state became aggravated, and finally large fungating growths developed, which in all their clinical features were malignant, and which caused the patient's death. Still, even to the end, the growths were neither epithelial nor sarcomatous, and they displayed a suceptibility of cure in some parts, whilst they developed in others. The cases which had been grouped together as examples of it were not all closely parallel, but they constituted connecting links, as it were, between inflammatory disorders and those of truly malignant character.

All that had been said as to granuloma fungoides applied with at least equal force to such maladies as pityriasis rubra, lichen scrofulosorum, lichen ruber, lichen planus, morphœa, and a whole host of others. In the departments of neurology and ophthalmology, also, new maladies of great rarity had recently been discovered and named, which reflected back most valuable light upon the domain of general pathology. The discovery, separation, and naming of new diseases was not, as a rule, accepted with much gratitude. Acromegaly might be taken as an example. Most people on first hearing of it had felt a profound hope that they might be spared any further acquaintance with an outlandish name for a disease probably of no consequence. Yet under this quaint name was designated a marvel calculated to excite the deepest sentiment of wonder. That it should be possible for a man's head, feet, and hands to simultaneously take on overgrowth, for his scalp to get thick, his skull thick, his face enlarged, and for his fingers and toes to double their natural dimensions, was indeed a pathological phenomenon of the most unlooked-for kind. Our annoyance with the mention of the name and the discussion of the disease named ceased as soon as we realized the thing. So it was with other instances —repulsion at first, gratitude afterwards. Without doubt, those who gave new names had much to answer for if they did it injudiciously. Needless and inconvenient change was reprehensible, but there was no avoiding the introduction of many new names into all departments of clinical observation. Confusion arose only when different things were called by the same name. In the future progress of clinical knowledge, it was unavoidable but that names must be multiplied. There was no objection to the use, for a time at least, of the name of the discoverer as a designation for the thing he had discovered.

It was the duty of all who gave names, whether to their own discoveries or to those of others, to afford to the profession the best possible means of identifying and distinguishing that which was named. As soon as such identification could be easily made, the new name ceased to be an annoyance, and it became a matter of pleasure to employ it.

The zeal for nominal diagnosis, the anxiety displayed, especially in dermatology, to place every case under some recognized name, sometimes brought to mind the story of the old woman who remarked that it was a thing to be thankful for that when Adam gave names to the animals he named them all right. There seemed sometimes to be almost a degree of forgetfulness that the nature of the disease was of far more real importance than its name, and that this might be recognized quite independently of the name in use, and possibly even better without it. Names very often had the effect of cramping our conceptions of the realities.

